# Fast-Versus Slow-Resorbable Calcium Phosphate Bone Substitute Materials—Texture Analysis after 12 Months of Observation

**DOI:** 10.3390/ma13173854

**Published:** 2020-09-01

**Authors:** Tomasz Wach, Marcin Kozakiewicz

**Affiliations:** Department of Maxillofacial Surgery, Medical University in Lodz, Pl. Hallera 1, 90-647 Łódź, Poland; marcin.kozakiewicz@umed.lodz.pl

**Keywords:** alveolar crest, augmentation, bone substitute material, intraoral radiograph, texture analysis

## Abstract

The development of oral surgery and implantology has led to the need for better and more predictable materials. Various substitute materials are now used for bone regeneration. The replacement of scaffolding material by new bone tissue is the most important condition. This study aimed to evaluate the effects of the resorbability of bone substitute materials during regeneration to the jawbone. The study included 88 patients during the 12-month follow-up. All the patients had undergone oral surgical procedures using two different substitute materials—Cerasorb (high-rate resorbable (β-tricalcium phosphate)) and Endobone (low-rate resorbable (hydroxyapatite)). Texture analysis was performed in intraoral radiographs, in which regions of interest were established for the bone substitute materials and reference bone. Five texture features were calculated, namely the sum average (SumAverg), entropy (Entropy), and three Harr discrete wavelet transform coefficients. This study revealed that all 5 features described the healing process well. Entropy was decreased in both cases with time; however, in Cerasorb cases, the texture feature values were very close to those of the reference bone after 12 months of healing (*p* < 0.05). The wavelet transform coefficient at scale 6 also showed that longitudinal objects appeared in implantation sites, similar to trabecular bone (*p* < 0.05) after 12 months of healing. The slow-resorbing material restored the structure of the alveolar crest better in terms of producing large objects similar to the components of a barrel bone image (wavelet coefficients), but required a longer time for reconstruction. The fast-resorbing material showed a texture image with a similar scattering of structures to that of the reference bone (entropy) after 12 months.

## 1. Introduction

The bone regeneration process is similar to the bone healing process that occurs after fractures or during the bone deficiency regeneration process. Three overlapping stages occur throughout the human lifespan—early inflammatory, repair, and remodelling. All three stages are influenced by hormones; cells; and biomechanical, biochemical, and pathological mechanisms. Other factors can also disturb these processes, such as smoking, using cytotoxic medication and anti-inflammatory drugs, or ingrowth of soft tissue [1,2,3].

The removal of retained teeth, standard dental extractions, apicoectomy, sinus lift, and pathological lesions in jawbones (e.g., cysts) cause bone deficiencies, which can be filled by bone substitute (BS) materials. Various BS materials (e.g., inorganic, natural, and synthetic polymers, as well as composites) are used for bone regeneration and to obtain optimal vertical and horizontal bone volume dimensions (e.g., before implantological or prosthetic treatment) [4]. For example, blocks of collagenated cancellous equine bone [5] are used in the vertical bone regeneration method. Additionally, biomaterials functionalised with proteins (e.g., fibronectin and BSP) increase the interactions with cells, leading to better osteoinductivity properties of the materials [6]; however, the gold standard is the use of autologous bone grafts.

Calcium phosphate (CP) materials have gained increased attention since the 1980s. The properties of CPs, such as their resorbability, biocompatibility, non-toxicity, osteoconductivity and osteoinductivity, immunological inertion, and non-teratogenic and non-carcinogenic characteristics, have led to their use in bone regeneration surgery procedures [7,8,9]. Some CP bone substitute materials show a faster resorption rate, while others show a slow resorption rate. If the resorbing level is high, the use of a titanium mesh can be useful to obtain the required bone volume after bone regeneration [10].

The resorbing properties mentioned above are crucial in bone regeneration processes. Due to the osteoclast function, bone substitute materials should be resorbed and replaced by new bone tissue. If the bone substitute material resorbs in adequate time, the bone tissue regenerates (ingrowth) faster and the bone quality is better than that in cases where resorption takes more time (or occurs too early) and new bone cannot fill the bone defects [9]. Scarano et al. showed that after 4 months of healing, the residual particles of biomaterial could be found histologically [5].

Two questions have arisen: Is it possible to investigate the microarchitecture of rebuilt bone substitute materials without additional surgical procedures? Is the new bone tissue similar to human bone tissue after a one-year observation period? Thus, this study aimed to analyse the textures of radiographs of high- and low-rate resorbable CP as bone substitute materials in alveolar crest augmentations.

## 2. Materials and Methods

The study was approved by the University Ethical Committee (RNN/485/11/KB). Eighty-eight patients (35 male and 53 female) aged between 10 and 72 years (average age: 45.21 years) were included. All the patients had undergone surgical procedures under local anaesthesia before dental implant insertion for either sinus lift (39 patients) or tooth extraction (49 patients). The patients were divided into 2 groups depending on the used material:

1. High-rate resorbable: β tricalcium phosphate (Curasan: Cerasorb M, Wake Forest, NC, USA);
2. Low-rate resorbable: hydroxyapatite (Zimmer Biomet Dental: Endobone, Palm Beach Gardens, FL, USA).

The inclusion criteria were two-dimensional radiographs after surgery and 12 months later. The exclusion criteria were defects in radiographs (in the visual assessment of authors) and circumstances requiring bone grafting. No exclusion criteria considered general diseases.

**Figure 1:**
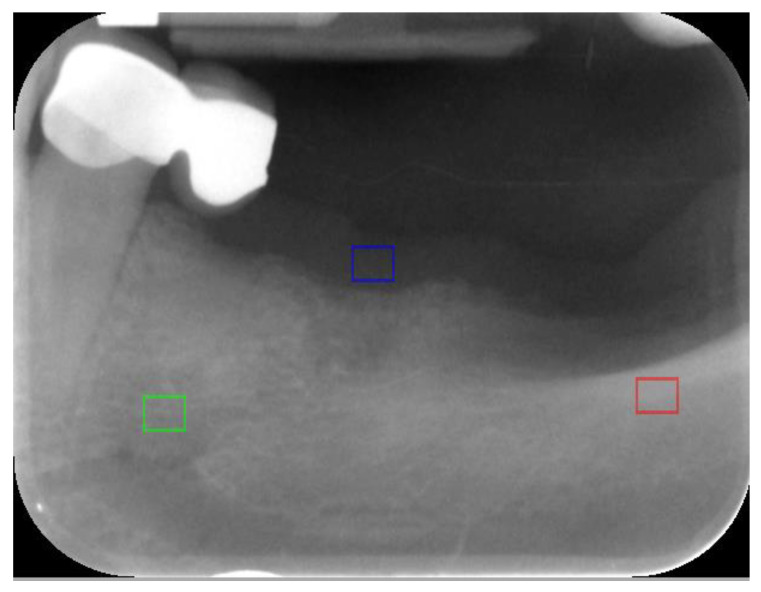
Regions of interest (ROI) marked for reference: cortical bone (red ROI), trabecular bone (green ROI), and soft tissue (gingiva) (blue ROI).

A preliminary investigation was performed to evaluate which values of the textural features describe the cortical bone, trabecular bone, and soft tissue as a reference. Thirty samples from each of these regions of interest (ROIs) were analysed (Figure 1).

All two-dimensional radiographs were acquired during typical clinical follow-up: on the day of surgery, directly after surgery (00 M), and 12 months (12 M) after surgery. The Digora Optime system (KaVo Dental, Brea, CA, USA) of radiography was used to acquire intraoral radiographs in a standardised way using strictly determined technical parameters: 7 mA and 70 mV in 0.1 s. Positioners were used to take radiographs repeatably (90° angle of the X beam to the surface of the phosphor plate).

All radiographs were analysed in MaZda software version 4.6, which was invented by the University of Technology in Lodz [11,12,13] The main goal of this software is texture analysis through quantitative description. It allows the evaluation of texture parameters in digital X-rays. In this study, 176 X-rays were analysed.

**Figure 2:**
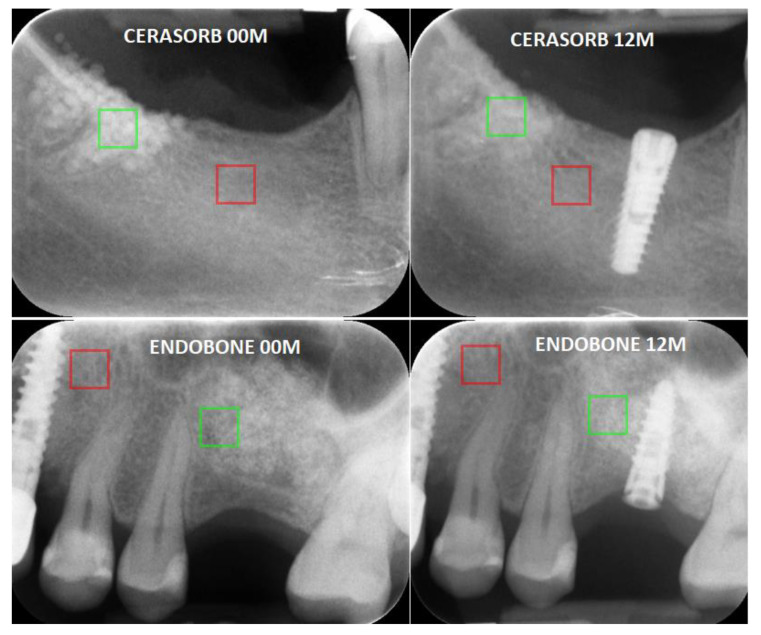
Examples of analysed intraoral radiographs (directly after implantation (00 M) and one year after surgery (12 M)). The exemplary regions of interest (ROI) are marked in green (material ROI) and red (bone ROI).

First, the X-ray image was imported to MaZda in the.bmp file format (bit map). Next, the ROIs were marked with an average of 2370 pixels for the bone area and 2541 pixels for the material area. ROIs greater than 800 pixels were found to be sufficient to perform repeatable texture analysis [13]. In all images, material texture ROIs were marked in green and reference bone ROIs were marked in red (Figure 2). The ROIs were normalised to share the same mean and standard deviation of the grey level inside the ROI (μ ± 3σ, where μ and σ denote the mean and standard deviation of the registered optical density, respectively).

The components of texture analysis were as follows: sum of squares (SumOfSqrs), sum average (SumAverg), entropy (Entropy), difference entropy (DifEntr) for a 5-pixel distance (all previous features derive from co-occurrence matrix), long run-length emphasis moment (LngREmph), and short run-length emphasis moment (ShrtREmph). Furthermore, the wavelet decomposition energy was analysed as a component of the texture (filtering in the horizontal and vertical directions LL, LH, HL, and HH, where L is low-pass filtering and H is high-pass filtering at three scales—4, 5, and 6) [14,15]. The features were calculated for the reference bone and substitute material as follows:

$$SumOfSqrs=\overset{N_{g}}{\underset{i=1}{\sum }}\overset{N_{g}}{\underset{j=1}{\sum }}{\left(i-\mu_{x}\right)}^{2}\;p\left(i,j\right)$$

$$Entropy=-\overset{N_{g}}{\underset{i=1}{\sum }}\overset{N_{g}}{\underset{j=1}{\sum }}p\left(i,j\right)\log\left(p\left(i,j\right)\right)$$

where Σ is the sum, N is the number of levels of optical density in the radiograph, *i* and *j* are optical densities of pixels at a 5-image-point distance from each another, *p* is the probability, and log is the logarithm [14].

$$DifEntr=-\overset{N_{g}}{\underset{i=1}{\sum }}p_{x-y}\left(i\right)\log(p_{x-y}\left(i\right))$$

$$LngREmph=(\overset{N_{g}}{\underset{i=1}{\sum }}\overset{N_{r}}{\underset{j=1}{\sum }}j^{2}p\left(i,j\right))/C$$

where Σ is sum, N is the number of series of pixels with a density level *i* and length *j*, Ng is the number of levels for image density (8 bits, i.e., 256 grey levels), Nr is the number of pixels in series, *p* is the probability, and C is the coefficient, as below:

$$C=\overset{N_{r}}{\underset{j=1}{\sum }}\overset{N_{g}}{\underset{i=1}{\sum }}p\left(i,j\right)$$

$$SumAverg=\overset{2N_{g}}{\underset{i=1}{\sum }}ip$$

where x and y are the coordinates (row and column) of entry in the co-occurrence matrix and *p* x + y is the probability of the co-occurrence matrix coordinates summing to x + y.

Next, the medians of SumOfSqrs, SumAverg, Entropy, DifEntr, LngREmph, ShrtREmph, and the Haar wavelet decomposition (LH, HL, LL, HH) were assessed for the reference bone and each material individually after each control period (i.e., 00 M and 12 M) were analysed statistically to describe the texture structure variability.

The Fisher coefficient for material and bone ROIs was also calculated: an ROI value greater than 1.0 indicates a significant difference between the material and bone ROIs throughout 12 months of observation. The higher the Fisher coefficient value (higher than 1), the larger the difference in the texture structure.

### Statistical Analysis

The Kruskal–Wallis test (to compare time-dependent alterations in medians) was applied for statistical analysis. Next, a multiple comparison procedure was used to determine which means were significantly different. The method discriminates among the variables in the Fisher’s least significant difference (LSD) procedure. The difference was considered significant if *p* < 0.05. Stargraphics Centurion XVI (StarPoint Technologies. INC., The Plains, VA, USA) was used for statistical analysis.

## 3. Results

Not all of the features were statistically significant (*p* < 0.05)—*p* < 0.05 was only observed for the following:

- Sum average;
- Entropy
- Wavelet energy (LL_s-4, LL_s-5, LH_s-6).

The preliminary characteristics evaluating radiotextural features were determined for the cortical bone, trabecular bone, and gingiva as a soft tissue reference.

The basal entropy reference in bone is presented in Table 1. The entropy feature indicates bone tissue (*p* < 0.05), whereby ROI values higher than 2.61 do not indicate soft tissue (Table 2). The SumAverg indicates cortical bone with a value higher than 63.14 (*p* < 0.05).

The wavelet decomposition for sub-band LL at scales 4 and 5 shows where cortical bone may be expected (*p* < 0.05):

- ROI values lower than 17,910 (approximately 10,674) for WavEnLL_s-4 and lower than 18,577 (approximately 6485.85) for WavEnLL_s-5 represent cortical bone;
- WavEnLH_s-6 indicates bone tissue that is better than other wavelets, while ROI values between 484.04 and 523.24 indicate bone tissue instead of soft tissue (Table 2).

The SumAverg for Cerasorb was significantly higher (*p* < 0.05) than that for the reference bone, showing 64.52 for the material at 00 M and lower (64.04) at 12 M (*p* < 0.05), which was still higher than that for the reference bone. The SumAverg for Endobone was also significantly higher (*p* < 0.05) than that for the reference bone, at 64.22 at 00 M, which decreased to 63.94 after 12 months, however still remaining significant (*p* < 0.05).

**Figure 3:**
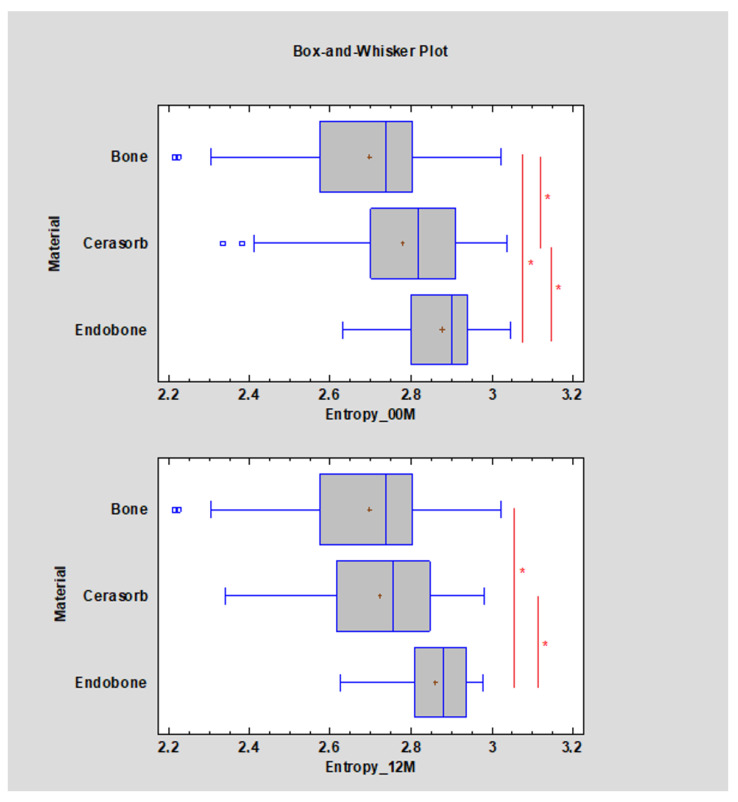
Bone-substitute-dependent alteration of the radiotexture defined by entropy in the implantation site. Observations immediately post-operation (00 M) and 12 months after surgery (12 M). Asterisks indicate statistically significant differences (*p* < 0.05).

The entropy for Cerasorb was significantly (*p* < 0.05) higher than that for the reference bone at 00 M: 2.82, decreasing to 2.76 (*p* < 0.05) towards the reference bone value. The entropy for Endobone was significantly (*p* < 0.05) higher than that for the reference bone at 00 M and decreased insignificantly to 2.88 with *p* < 0.05 at 12 M. The multiple range test for entropy did not reveal differences between Cerasorb and the reference bone after 12 months (*p* < 0.05) (Figure 3).

Values for wavelet decomposition:

- The WavEnLL_s-4 value at 00 M for Cerasorb was 16,885, while the value for Endobone was 17,657. After 12 months the WavEnLL_s-4 value for Cerasorb was 15,876 (it decreased *p* < 0.05), while that for Endobone was 17,166 (*p* < 0.05);
- The WavEnLL_s-5 (*p* < 0.05) value at 00 M for Cerasorb was 16,073, while that for Endobone was 18,690. After 12 months the WavEnLL_s-5 value for Cerasorb was 15,907, while that for Endobone was 19,292 (*p* < 0.05);
- The WavEnLH_s-6 values after 12 months were 338.47 for Cerasorb and 231.82 for Endobone (*p* < 0.05).

**Figure 4:**
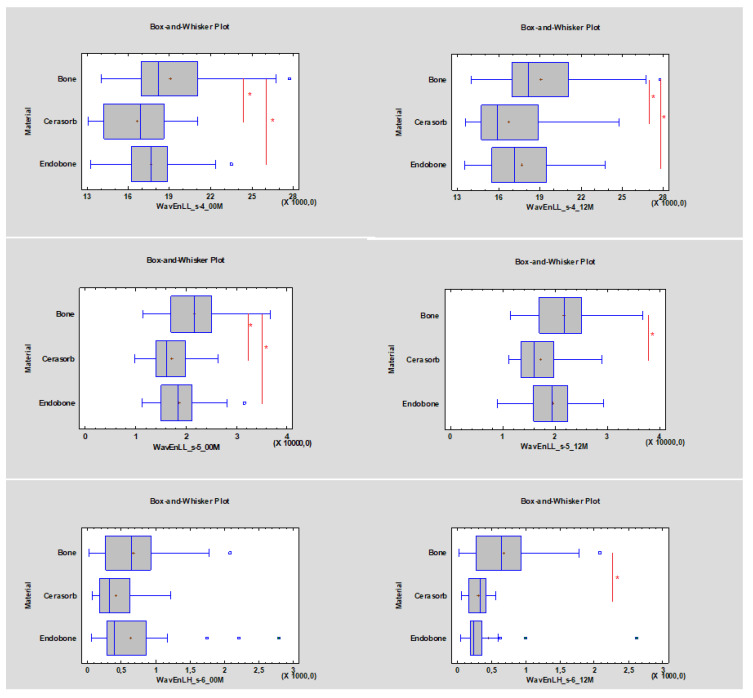
Bone-substitute-dependent alteration of the radiotexture defined by the wavelet energy decomposition in the implantation site. Observations immediately post-operation (00 M) and 12 months after surgery (12 M). Asterisks indicate statistically significant differences (*p* < 0.05). The reference is the trabecular bone.

The WavEnLL_s-4 value decreased for both materials during the observation period and became more similar to the bone reference value for Cerasorb. Considering that WavEnLL_s-5 showed that the Cerasorb value was similar to the reference bone value with *p* < 0.05, the WavEnLH_s-6 value (indicating bone) also became more similar to the value range of the reference bone, ranging between 484.04 and 523.24 after 12 months of the healing process for Cerasorb compared with that for Endobone (Figure 4).

ANOVA for Cerasorb after 12 months of healing showed a similar value as that for the reference bone when considering the SumAverg feature (*p* < 0.05) (Table 3).

## 4. Discussion

Before prosthetic treatment, dentists must determine the required bone volume. Autologous bone grafts remain the gold standard in bone regenerative surgery. Only autologous bone grafts have a regeneration triad and have several other advantages, such as short healing times, favourable bone quality, and predictability in large-defect regeneration [16]. Bone grafts are not always feasible and surgeons often use bone substitute material before implantological treatment to obtain the required volume of the alveolar crest dimension [17].

Before implantation, it is not possible to check whether the healed and regenerated tissue is similar to that of the bone tissue without surgical intervention and histological evaluation [18]. What if the tissue structure could be analysed based on two-dimensional radiographs without surgical intervention? In 1973, Haralick et al. were the first to present the textural features for image classification. These features were based on grey-tone spatial dependencies [19]. These authors proposed a method whereby neighbouring pixels in an image were analysed. These methods, applied in MaZda texture features (second-order features), can be divided into several groups, including by histogram analysis, mean, variance, skewness, kurtosis, and percentiles (first-order features):

- Co-occurrence matrix derived: angular second moment, contrast, correlation, sum of squares, inverse difference moment, sum average, sum variance, sum of entropies, entropy, difference variance, and difference entropy;
- Run-length matrix derived: run length nonuniformity, grey level nonuniformity, long run emphasis, short run emphasis, and fraction of image in runs.

There is also the possibility of evaluating wavelet parameters [20] that were analysed in this study.

Entropy is a measure of checking tissue microstructure irregularity. Kołaciński et al. showed that changes in entropy could be related to bone defects in the healing process [21]. This texture feature is useful for the follow-up of studies, such as in the present study. Additionally, Kozakiewicz et al. revealed that the energy of the wavelet decomposition is useful for the mathematical radiotextural evaluation of the bone microstructure [22]. In this study, the texture features were used and the bone healing process was investigated.

Bone substitute materials in the healing period transit through the resorption process. The resorption process leads to the degradation of material granules and the ingrowth of living bone [23]. The resorption rate depends on many factors, including the microporosity and chemical structure of the substitute material (ion content). It was proven that if the material mainly comprises hydroxyapatite rather than calcium phosphate then the resorbability of this material will be lower. Resorption is also dependent on the crystallinity and local biological environment [24,25]. Cerasorb, comprising β tricalcium phosphate, shows a higher rate of resorption than Endobone, comprising hydroxyapatite, based on texture features analyses in this study.

The difference in the surface properties affecting the resorption and adhesion of osteogenic cells is called the microporosity of the material granules. Bone ingrowth into the bone substitute material during the healing process is correlated with microporosity [26]. Cerasorb is characterised by granule dimensions in the range of 1000–2000 μm, with pores ranging from 5 to 500 μm. It is a pure high-rate resorbable β tricalcium phosphate. The dimensions of Endobone granules are in the range of 500 to 2000 μm, with pores ranging between 150 and 550 μm. It is made of low-rate resorbable hydroxyapatite [27]. Our study confirms that the Cerasorb material healing process is faster than Endobone, as shown by texture analysis. We can also conclude that a bone substitute material with small micropores regenerates faster than one with larger pores in the material granules.

Texture analysis can detect a value change of a feature due to a change in the radiolucency of the material in an X-ray image. The resorption process changes the transparency of the regenerated tissue [28]. Our study shows that a higher value of entropy in 00 M for both types of BS is related to the presence of low-radiolucent granules in the used material; through months of observation, the entropy values decreased towards that of the reference bone. After 12 months of healing, the microstructure of the Cerasorb bone substitute material represented bone tissue better than Endobone.

Replacement of the substitute material with living bone is the main condition of bone regeneration. This stage of bone healing can be partial or almost complete [23,29]. Some review articles have reported that the phased resorption of material leads to systematic new bone formation [30]. High-rate resorbable materials may be replaced by new bone faster than those with low-rate resorbability. However, the materials characterised by a low level of resorbability maintain the dimensions of the regenerated region and can lead to better clinical results [26]. Cerasorb resorption occurs faster and the regenerated tissue is similar to that of the reference bone. Endobone material also resorbs well, but it needs more time to complete the transformation into new bone tissue.

The texture analysis can lead to interesting conclusions concerning wavelets. The decomposition of wavelets can lead to interesting observations depending on the values of the wavelet scale. The higher the scale (4, 5, or 6 appears in the image texture), the larger the objects in the texture analysis appear. Sub-band LL indicates smaller objects than HH; however, in both cases, they have circular shades. Sub-bands LH and HL indicate longitudinal objects.

Decreasing values (from 4 and 5 in relation to the 6 scale) may indicate the disappearance of small objects and the formation of larger ones, indicating a progressive healing process, resorption of material granules, and the appearance of trabeculae [22]. Wavelets at scale 6 in sub-band HL in the case of Cerasorb may lead to the conclusion that more longitudinal objects, similar to large trabecular ones, or the foci of sclerotisation appear in the image texture. Thus, Cerasorb material is more similar to the reference bone tissue. The decomposition of wavelets in Endobone reveals the requirement for a longer observation period to identify alterations in the slow resorbable material.

## 5. Conclusions

Regarding the radiological images of the two phosphate compounds, the slow-resorbing one restores the alveolar crest better but requires more time for recovery. The fast-resorbing material, after 12 months of observation, becomes more similar to the reference bone tissue and is a promising BS because it directs the structural alteration to vital bones. The limitations of the study are that all the analyses were based on X-ray data and the study lacks a comparison to histological samples.

## Figures and Tables

**Table 1:** Report on the entropy change during the period of the experiment for cancellous and cortical bone.

Reference Region	00 M	12 M	Significance
Cancellous bone	2.69 ± 0.18	2.71 ± 0.18	*p* = 0.585
Cortical bone	2.55 ± 0.10	2.48 ± 0.21	*p* = 0.542

Abbreviations: 00 M—data acquired at the start of the experiment; 12 M—data acquired at the end of the experiment; *p*—the probability of obtaining test results at least as extreme as the results actually observed, under the assumption that the null hypothesis is correct.

**Table 2:** Values of the texture features in the reference bone.

Texture Feature	Value	*p* Value	Reference
Entropy	<2.61	*p* < 0.05	Bone tissue (cortical and cancellous)
SumAverg	>63.14	*p* < 0.05	Cortical bone
Wavelet LL_s-4	<17,910 (approximately 10,674)	*p* < 0.05	Cortical bone
Wavelet LL_s-5	<18,577 (approximately 6485)	*p* < 0.05	Cortical bone
Wavelet LH_s-6	484–523	*p* < 0.05	Bone tissue (cortical and cancellous)

Abbreviations: SumAverg—sum of averages between pixel optical densities in investigated distans; Wavelet LL_s LH_s—the Haar wavelet decomposition and coefficient energy after lowpass filter (L), highpass filter (H), and in given scale (s-); *p*—the probability of obtaining test results at least as extreme as the results actually observed, under the assumption that the null hypothesis is correct.

**Table 3:** Fisher’s coefficient values for Cerasorb and Endobone materials. A value higher than 1.0 indicates a higher difference between the bone and substitute material.

Fisher’s Coefficient	FOLLOW-UP	
Material	00 M	12 M
Endobone	S(0,5)SumAverg 1.1	S(0,5)SumAverg 1.6
	S(0,5)Entropy 1.4	S(0,5)Entropy 1.2
	S(5,5)Entropy 1.2	S(5,5)Entropy 1.0
	S(5,5)Entropy 1.2	S(5,5)Entropy 0.9
	S(5,0)Entropy 1.1	S(5,0)Entropy 0.9
Cerasorb	S(0,5)SumAverg 1.8	S(0,5)SumAverg 1.4
	S(0,5)Entropy 0.9	S(0,5)Entropy 0.6
	S(5,5)Entropy 0.8	S(5,5)Entropy 0.5
	S(5,5)Entropy 0.8	S(5,5)Entropy 0.5
	S(5,0)Entropy 0.7	S(5,0)Entropy 0.4

Abbreviations: 00 M—data acquired at the start of the experiment; 12 M—data acquired at the end of the experiment; SumAverg—sum of averages between pixel optical densities in investigated distance.
